# Cost-effectiveness of second-line therapies in adults with chronic immune thrombocytopenia

**DOI:** 10.1002/ajh.26497

**Published:** 2022-02-24

**Authors:** George Goshua, Pranay Sinha, Natalia Kunst, Lauren Pischel, Alfred Ian Lee, Adam Cuker

**Affiliations:** 1 Section of Hematology, Yale University School of Medicine, New Haven, Connecticut, USA; 2 Department of Health Policy and Management, Harvard T.H. Chan School of Public Health, Boston, Massachusetts, USA; 3 Section of Infectious Diseases, Boston Medical Center, Boston, Massachusetts, USA; 4 Department of Population Medicine, Harvard Medical School and Harvard Pilgrim Healthcare Institute, Boston, Massachusetts, USA; 5 Section of Infectious Diseases, Yale University School of Medicine, New Haven, Connecticut, USA; 6 Department of Epidemiology of Microbial Diseases, Yale School of Public Health, New Haven, Connecticut, USA; 7 Department of Medicine and Department of Pathology & Laboratory Medicine, Perelman School of Medicine, University of Pennsylvania, Philadelphia, Pennsylvania, USA

## Abstract

Major options for second-line therapy in adults with chronic immune thrombocytepenia (ITP) include splenectomy, rituximab, and thrombopoietin receptor agonists (TRAs). The American Society of Hematology guidelines recommend rituximab over splenectomy, TRAs over rituximab, and splenectomy or TRAs while noting a lack of evidence on the cost-effectiveness of these therapies. Using prospective, observational, and meta-analytic data, we performed the first cost-effectiveness analysis of second-line therapies in chronic ITP, from the perspective of the U.S. health system. Over a 20-year time-horizon, our six-strategy Markov model shows that a strategy incorporating early splenectomy, an approach at odds with current guidelines and clinical practice, is the cost-effective strategy. All four strategies utilizing TRAs in the first or second position cost over $1 million per quality-adjusted life-year, as compared to strategies involving early use of splenectomy and rituximab. In a probabilistic sensitivity analysis, early use of splenectomy and rituximab in either order was favored in 100% of 10 000 iterations. The annual cost of TRAs would have to decrease over 80% to begin to become cost-effective in any early TRA strategy. Our data indicate that effectiveness of early TRA and late TRA strategies is similar with the cost significantly greater with early TRA strategies. Contrary to current practice trends and guidelines, early use of splenectomy and rituximab, rather than TRAs, constitutes cost-effective treatment in adults with chronic ITP.

## 1 | INTRODUCTION

Immune thrombocytopenia (ITP) is an autoimmune disorder characterized by accelerated platelet clearance and impaired platelet production. ITP is termed “chronic” when it has been present for at least 12 months.^1^ Relative to the pediatric population, spontaneous remission of ITP in adults is less common, with over half of affected adults developing chronic ITP.^2^

First-line treatments for ITP include corticosteroids and intravenous immune globulin.^1^ However, these treatments are not suitable for long-term use. Therefore, adults with chronic ITP often require second-line therapy to maintain a hemostatic platelet count. The three major second-line therapies for ITP include the anti-CD20 monoclonal antibody rituximab; the thrombopoietin receptor agonists (TRAs), romiplostim, eltrombopag, and avatrombopag; and splenectomy.^1^ The 2019 American Society of Hematology (ASH) guidelines recommend (1) TRA over rituximab (recommendation #9) and (2) rituximab over splenectomy (recommendation #8), with splenectomy being the least favored treatment. As a reflection of the ASH guidelines, the use of splenectomy in adult patients with chronic ITP is often reserved for patients who fail TRA or rituximab therapy. The guidelines note that these three treatments are often considered simultaneously and that treatment should be selected on an individual basis based on patient values and preferences, availability of therapies, and cost.^1^

Despite their vastly different durable response rates, toxicity profiles, and costs, cost-effectiveness of second-line therapies for chronic ITP has not yet been examined. This is notable given the impact of rare diseases on the healthcare system, where costs are disproportionate to disease prevalence.^3,4^ We sought to address this knowledge gap by conducting the first cost-effectiveness analysis of second-line treatment strategies in chronic ITP.

## 2 | METHODS

### 2.1 | Overview of model

We created a Markov model that examined the six possible treatment strategies incorporating different sequences of the three major second-line therapies currently used in clinical practice to treat chronic ITP: rituximab, TRAs, and splenectomy.^1^ Each strategy represents a combination of up to three treatments administered in a different sequential order (Figure S1). For each treatment strategy, the patient proceeds with the initial treatment unless and until it is no longer effective, then transitions to the second therapy in the sequence unless and until is it no longer effective, and then proceeds to the third and final treatment in the sequence. If a treatment is effective, the patient does not proceed to the next treatment. We assumed that the only reason for changing treatments was ineffectiveness and that no changes occurred due to toxicity, cost, or other factors. Of all six possible treatment strategies, #1–4 are consistent with the 2019 ASH guidelines and #5–6 are not (Figure S1). We constructed our model using TreeAge Pro Healthcare 2021 (TreeAge Software).

### 2.2 | Model assumptions: Overall

In our Markov model, we assumed a starting age of 51 years. This assumption is based on the median age at diagnosis of ITP of 50 years and the recommendation to avoid splenectomy in the first year after diagnosis because spontaneous remission may be most likely during this time period.^5^ Transition-state cycles were 12 months in duration, with a total run of 20 cycles (i.e., 20-year time-horizon) to estimate the expected benefits and costs at a time-point where there is data for effectiveness of splenectomy in chronic ITP. Annual background mortality probabilities from other, non-ITP causes were assumed to be the same as found in the general population and were obtained from the Social Security Actuarial Life Table.^6^ The background mortality was weighted to reflect that males in the general population have higher background all-cause mortality probability and that females disproportionately represent the ITP patient population with roughly 72% of ITP diagnoses up to the age of 60.^2^ Base case estimates and ranges for all input parameters used in the model and for sensitivity analyses are reported in Table 1.

### 2.3 | Model assumptions: Splenectomy

We made several conservative assumptions to avoid overestimating the effectiveness of splenectomy. Complete response rates to splenectomy, defined as achieving a platelet count >150 × 10^9^/L, observed at 5-, 10-, and 20-year follow-up range from 60% to 88.5%.^10,12–15^ We utilized the lower estimate of 60% and further decreased and increased this estimate by 20%, corresponding to as low as 48% and as high as 72% in our sensitivity analyses.^10,12–15^ In addition, we utilized only complete response rates for splenectomy while using less stringent overall response rates (platelet count >30–50 × 10^9^/L) for rituximab and TRAs.

We also utilized upper-bound estimates for postsplenectomy complications in patients with ITP. These included an annual probability of thrombosis (both venous and arterial) of 1.7% that we assumed remained constant for all 20 years in our Markov model despite annualized rates that are lower than this estimate and despite recent evidence that 5-year arterial risk (inclusive of myocardial infarction, pulmonary arterial hypertension, and stroke) in patients with ITP following splenectomy is not significantly increased as compared to age- and gender-matched controls.^18,22,31–34^ Historic rates of postsplenectomy infection requiring hospitalization still include data from prior to the introduction of the 13-valent pneumococcal conjugate vaccine and are highest in the septuagenarian decade at 13.85 per 100 person-years.^17,35^ Even though lower infection rates have been reported in the fifth decade (7.46 per 100 person-years), where our Markov model starts at a median age of 51, we utilized the higher septuagenarian rate and assumed every hospitalization was for the most severe sepsis state of septic shock, rather than for sepsis or severe sepsis. Furthermore, although the rate of overwhelming postsplenectomy infection is reported at 0.13 per 100 person-years and the mortality range is 50%–70%, we assumed the higher mortality of 70% as our base case.^16^ We accounted for the possibility of accessory splenectomy as previously reported in 9% of cases, assumed that all redo splenectomies would result in ITP treatment failure (i.e., 0% success probability), and assumed all thrombotic sequelae following splenectomy would require indefinite anticoagulation.^11^ We assumed a perioperative mortality rate for laparoscopic splenectomy of 0.2%, rather than that for open splenectomy (1.0%), as the former is the standard-of-care in the United States, although in our sensitivity analyses we examined the effect of open splenectomy mortality of 1.0%.^10^

### 2.4 | Model assumptions: Rituximab

For 1 cycle of rituximab therapy, we modeled the overall response rate at 38%–62.5%, 31.0%–33.3%, and 21% at 1, 2, and 5 years, respectively (Table 1).^24–27^ We assumed that rituximab was perfectly tolerated with no toxicities such as hepatitis B reactivation or progressive multifocal leukoencephalopathy, that patients who achieved an overall response at 5 years continued to have an overall response through to the conclusion of the model, and that there was no retreatment with rituximab.

### 2.5 | Model assumptions: TRA

Given similar 5-year overall response rates and costs between the TRAs romiplostim and eltrombopag,^22^ we treated these agents as interchangeable. Given that avatrombopag is a newer TRA and is available in fewer jurisdictions, we did not incorporate it in our analysis. For TRAs, we utilized an initial overall response rate of 80% and assumed a best case scenario where this response is sustained in all patients throughout the duration of the Markov model, despite rates of persistent response reported as low as 40%–50%.^29^ We further increased the response rate to TRA therapy by 20%, to 96% in our sensitivity analyses. We did not model adverse effects such as thrombosis, assuming perfectly tolerated TRA therapy. We also accounted for successful discontinuation of TRA therapy in 28% of individuals with chronic ITP after 2 years of therapy, as has been reported at a median treatment-free follow-up of 2.8 years.^30^ We assumed that such patients successfully coming off TRA therapy would live the rest of their lives without relapse or need for retreatment and would no longer accrue the costs of TRA therapy.

### 2.6 | Quality-adjusted life years

Health outcomes estimated by our model were expressed in quality-adjusted life years (QALYs), a measure that accounts for both health-related quality of life and length of life. QALYs in ITP have been previously measured in a number of different studies as reported in the Cost Effectiveness Analysis Registry at Tufts University (http://healtheconomicsdev.tuftsmedicalcenter.org/cear2/search/search.aspx). Reflecting these, we opted for the lowest value for the weighted utility of the ITP disease state of 0.74 and the highest value for the ITP “well” state of 0.987 in our base case.^7^ We selected these for the base case as it is expected to be most favorable for TRA-early treatment strategies.

### 2.7 | Costs

All costs were estimated in 2020 US Dollars.^36^ For cost of TRAs, we used the price of $113 976 yearly, as both previously determined and calculated from GoodRx based on the average wholesale price for either romiplostim or eltrombopag and assuming average dosing of 3 μg/kg per week and 50 mg daily, respectively.^14^ For rituximab therapy, we used the US average wholesale price at lymphoma-dosing as previously reported for a 170 cm, 70 kg individual ($8151 per dose for 4 doses per cycle).^28^ Our sensitivity analyses encompassed the cost of biosimilars (Truxima and Ruxience), which are less expensive than rituximab.^37^ Cost of laparoscopic splenectomy included a median 4-day hospital stay and was obtained from Healthcare Bluebook as a Fair Price for New Haven, CT ($20 449) and is similar to previously reported costs.^14^ To this cost we added the cost of routine vaccination against *Streptococcus pneumoniae* (1 dose Prevnar and 2 doses Pneumovax), *Haemophilus influenzae* type b (1 dose Acthib), and *Neisseria meningitidis* (2 doses MenACWY) ($828 total, available on GoodRx). The cost for accessory spleen evaluation included the cost of radionuclide imaging with a liver-spleen scan ($1165, available on MDSave) and repeat splenectomy in 9% of patients as previously reported in the setting of ITP.^11^ We used the most expensive sepsis related admission, that of septic shock ($42 285).^23^ For treatment of postsplenectomy thrombosis, we utilized the annual cost of apixaban and assumed an indefinite anticoagulation duration with a corresponding annual cost of $6000, as obtained from GoodRx.

### 2.8 | Cost-effectiveness analysis

We performed a cost-effectiveness analysis from a healthcare sector perspective.^38^ Both cost and health outcomes were appropriately discounted by 3% annually.^9^ We measured the cost-effectiveness of the treatment strategies considered by estimating the incremental cost-effectiveness ratios (ICERs), representing the difference in total costs divided by the difference in effectiveness associated with each pairing of the six treatment sequences considered. We started with ordering the strategies by their costs in ascending order. Next, we identified and excluded strongly and weakly dominated strategies. A treatment strategy was deemed strongly dominated when, by definition, it was associated with higher costs and fewer QALYs relative to the next less costly strategy. A treatment strategy was deemed weakly dominated when, by definition, a combination of two alternative strategies was associated with improved costs and benefits. Finally, we determined a cost-effective strategy using a willingness-to-pay threshold of $195 300 per QALY, derived from the 2019 per capita U.S. gross domestic product, as previously reported.^39^ We also report the net monetary benefit of each strategy.^40^ The net monetary benefit is calculated by multiplying the effectiveness in QALYs by the willingness-to-pay and subtracting the cost of the given strategy.^40^

### 2.9 | Sensitivity analyses

To examine how sensitive our results are to parameter uncertainty for each treatment strategy, we performed one-way sensitivity analyses and scenario analyses to evaluate the impact of specific input parameters and assumptions on our results. Parameters were varied through a range of ±20% of the base case estimates or across previously reported estimates where data were available. For example, while in our base case analysis we assumed a 60% complete remission rate following splenectomy (literature range of 60%–88.5%), we examined the impact of changing the value of this parameter across the entire range of 60% ± 20% (48%–72%) on our ICER result. An exception to this range included perioperative splenectomy mortality, where we allowed the mortality to increase 500% from laparoscopic splenectomy (0.2%) to include the reported mortality for open splenectomy (1.0%) and the range of the utility values of the well state (0.900–0.987), which cannot decrease below that of the range of the utility of disease (range: 0.592–0.888) (Table 1). All treatment pathways were iteratively compared to the treatment strategy identified as cost-effective. We present all parameters affecting the ICER at least 10% in either direction.

Furthermore, we propagated the uncertainty in the input parameters to the outcomes of our Markov model by performing a probabilistic sensitivity analysis (PSA). To perform the PSA, we assigned appropriate probability distributions to all input parameters in our model. For clinical probabilities and health utilities, we used beta (β)-PERT distributions. For costs, we used gamma (γ) distributions. Subsequently, we varied all model parameters within their distributions by simulating 10 000 Monte Carlo iterations.

### 2.10 | Role of funding source

The study was funded by the Yale Forget Scholarship. The funding source had no role in the design, conduct, or interpretation of the study or the preparation, review or approval of the manuscript.

## 3 | RESULTS

### 3.1 | Base case cost-effectiveness analysis

The estimated cost, QALYs and net monetary benefits associated with each of the six treatment strategies at a 20-year time-horizon are reported in Table 2. Using the assumed willingness-to-pay threshold, we found that treatment strategy #5 (splenectomy followed by rituximab and then TRA therapy) represented the cost-effective treatment strategy with a total cost of $376 350, accrual of 12.08 QALYs and a net monetary benefit of $1 983 417. In order of decreasing net monetary benefits and decreasing favorability, the next strategies in order are #4 (rituximab followed by splenectomy and then TRA therapy), #1 (splenectomy followed by TRA and then rituximab), #3 (rituximab followed by TRA and then splenectomy), #6 (TRA followed by splenectomy and then rituximab), and #2 (TRA followed by rituximab and then splenectomy). Of these strategies, #3 and #2 were weakly and strongly dominated, respectively. All four treatment strategies that utilized TRA in the first or second position had an ICER of over $1 million or were dominated. While the cost-effective strategy accrued the least QALYs, the range in QALYs across all six strategies of 0.22 is much less substantial than the range of costs across the strategies of $687 066.

### 3.2 | Sensitivity analyses

In one-way deterministic sensitivity analyses, for all treatment pathways in which TRA was used first or second, no variation of parameter values reduced the ICER to less than $1 million (Table S1). Even reduction in the probability of complete response after splenectomy to 48% did not bring the ICER below this threshold. One-way sensitivity analyses comparing the cost-effective strategy #5 (splenectomy followed by rituximab and then TRA) with strategy #4 (rituximab followed by splenectomy and then TRA) showed that increasing splenectomy mortality probability from the laparoscopic approach (0.2%) to the open (1%) had the greatest effect on the ICER, decreasing the ICER for strategy #4 from $456 109 to $237 801, a value that remained above the prespecified willingness-to-pay threshold. The annual cost of TRAs would need to decrease over 80%, from $113 976 to $21 005, for any TRA-early strategy to have a net monetary benefit that surpasses the cost-effective strategy.

**FIGURE 1:**
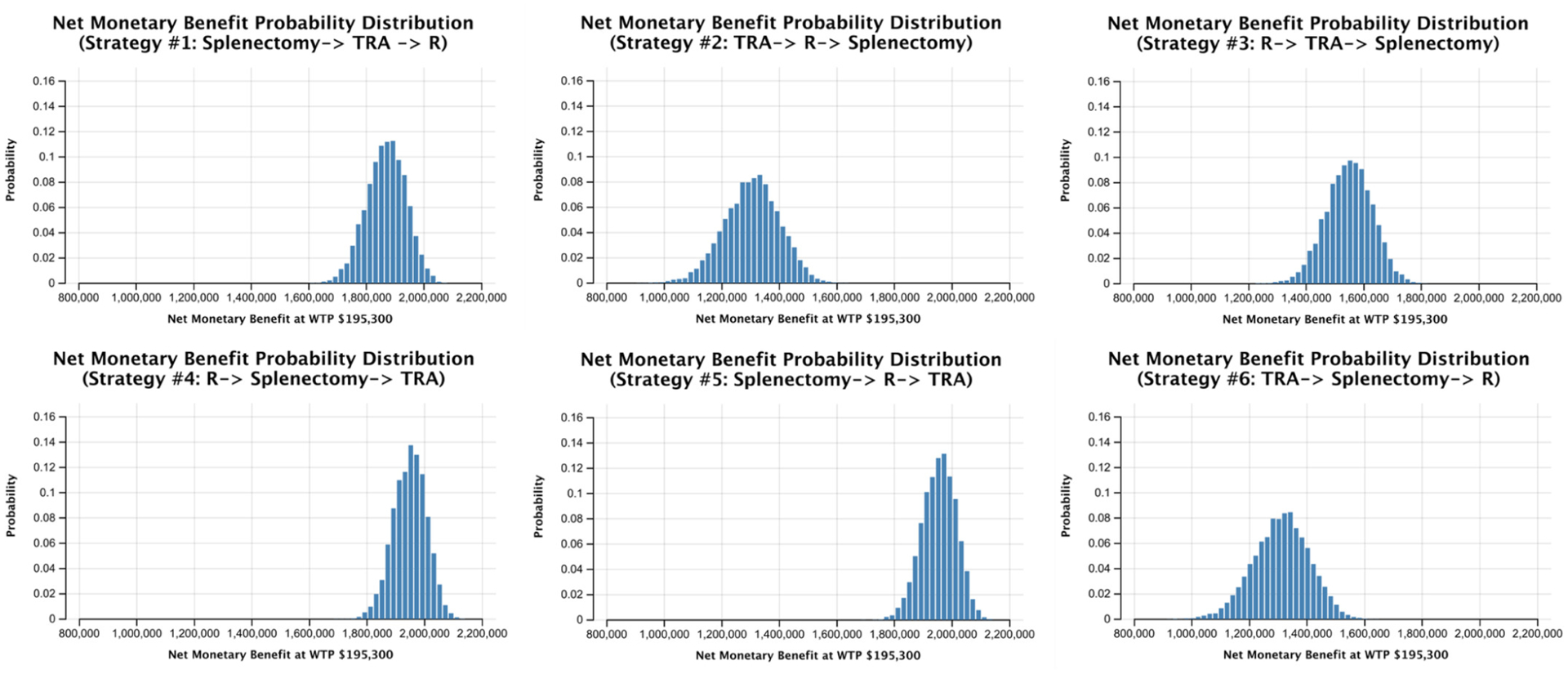
Probability histograms of net monetary benefit for strategies #1–6 in probabilistic sensitivity analysis. R, rituximab; TRA, thrombopoietin receptor agonist; WTP, willingness-to-pay

**FIGURE 2:**
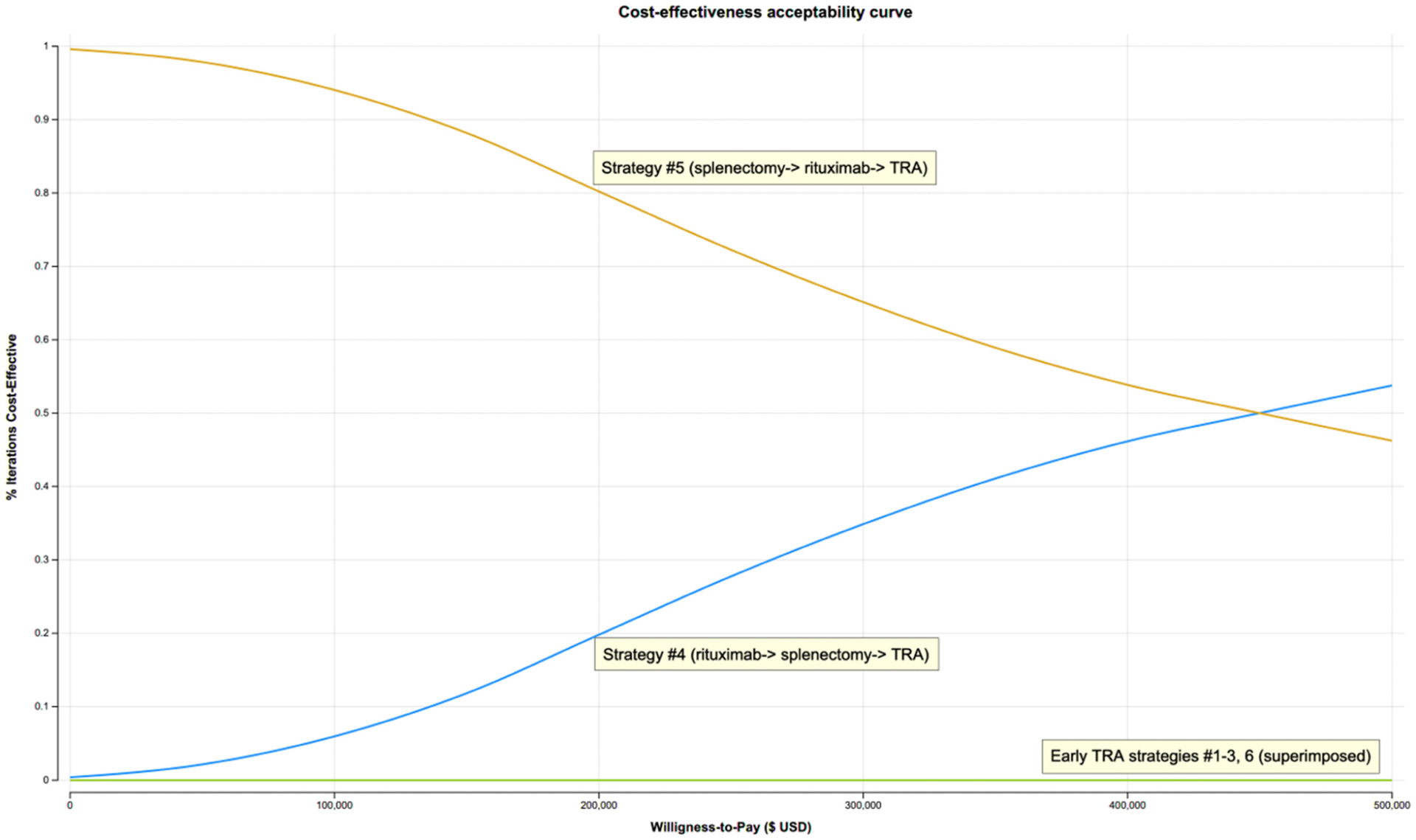
Cost-effectiveness acceptability curve. At a willingness-to-pay of $195 300, strategy #5 is favored in 81% and strategy #4 in 19% of 10 000 iterations. Early TRA therapy strategies (#1–3, 6) are favored in 0% of 10 000 iterations. TRA, thrombopoetin receptor agonist

The probability distributions of PSA net monetary benefits for each strategy visually capture the minimum and maximum net monetary benefit of each strategy, with a distinct disadvantage seen in early TRA strategy pathways (Figure 1). Furthermore, the cost-effectiveness acceptability curves presenting the PSA results show that at our assumed willingness-to-pay of $195 300/QALY, strategy #5 had the highest probability of cost-effectiveness of 81%, while strategy #4 comprised the remaining 19% (Figure 2). Early TRA strategies, where TRA is utilized first or second (i.e., strategies #1–3 and 6), were favored in 0.0% of 10 000 iterations in a Monte Carlo simulation.

## 4 | DISCUSSION

We examined the cost-effectiveness of the three major second-line therapies recommended in the 2019 ASH and International Working Group guidelines for treatment of chronic ITP. Our findings suggest that out of the six possible treatment strategies for patients with chronic ITP at a willingness-to-pay of $1 95 300/QALY, strategy #5 (splenectomy followed by rituximab and then TRA) represents the cost-effective strategy. This is notable for two reasons. First, strategy #5 is one of two strategies examined in our study that is at variance with ASH guidelines. Second, while the 2019 ASH guidelines suggest TRAs over rituximab and equivalently suggest TRA therapy or splenectomy, the ICERs for the treatment pathways that incorporate TRAs as the first or second sequential therapy do not represent a cost-effective strategy as they either substantially exceed the willingness-to-pay threshold, or are dominated.

Therapy with TRAs has several advantages, which have led to their use in second-line therapy and their endorsement in the 2019 ASH guidelines. They are associated with high overall response rates that tend to be sustained as long as the medication is taken, they are well-tolerated, and a up to a third of patients may be able to stop their TRA and maintain a response off all therapy. Despite these advantages, we found that early use of TRAs in second-line therapy is cost-ineffective, largely due to the high cost of the drugs and the need for ongoing therapy in most patients. In contrast, we found that early use of splenectomy in the second-line was cost-effective, largely because of high response rates without need for ongoing therapy in most patients. This held true despite our conservative assumptions, which tended to exaggerate risks associated with splenectomy and favor medical over surgical therapy, and across a variety of sensitivity analyses that covered the range of uncertainty present in all estimates, to ensure confidence in our conclusion. Furthermore, we identify that an annual price reduction of more than 80% in TRAs is warranted if any strategy with early TRA utilization is to begin to become cost-effective.

Limitations of our study include the varying quality of evidence with splenectomy response rates largely based on observational data, which we addressed in sensitivity analyses; assumption of equal probability of hemorrhagic complications across the six strategies, and applicability to patients who have had ITP for 1 year (i.e., chronic ITP) while noting that second-line therapy is often initiated prior to 1 year. We did not include other treatments not recommended as second-line therapy by the ASH guidelines, but that may still be used in clinical practice, such as mycophenolate mofetil and fostamatinib. For drug cost we did not consider the cost of an infusion chair for rituximab or romiplostim by design to further favor medical therapy. We examined cost-effectiveness of second-line therapy at the population level. By no means should our findings be interpreted to suggest that early splenectomy is appropriate for all individual patients because our model does not account for individual risk factors nor for patient preference. In a future study, individual risk factors can be built into a microsimulation model to examine effectiveness of treatment strategies across different cohorts of patients with chronic ITP. In addition, patient and physician preference is not captured in a cost-effectiveness analysis and may benefit from the development of a decision aid in this space.

In summary, we performed the first cost-effectiveness analysis evaluating second-line therapies for chronic ITP. We found that a strategy not recommended by the current ASH guidelines, that of splenectomy followed by rituximab and then TRA, represents the cost-effective strategy. Patients and clinicians have multiple options for second-line treatment of ITP including splenectomy, rituximab, and TRAs. These options vary in their response rates, side effect profile, and cost. The optimal treatment may be different for different patients depending on age, comorbidities, prior history, and patient values and preferences. Our cost-effectiveness analysis suggests that, for many patients, early use of splenectomy, rather than TRA, provides the best value.

## Supplementary Material

Supplemental Material

## Figures and Tables

**TABLE 1:** Base case input Parameters and probability distributions

Result or transition	Input parameters	Probability distribution	Study or data source
Cohort age at start, median in years	51	-	Grimaldi-Bensouda et al.^5^
Utility of ITP disease state	0.74 (0.74–0.89)	β-PERT (0.592, 0.888)	Kunz et al.^7^
Utility of ITP well state	0.987	β-PERT (0.90, 0.987)	Kunz et al.^7^
Annual health expenditure cost	$7650	γ (25, 306)	2016 MEPS Expenditures^8^
Discount rate	0.03	-	Sanders et al.^9^
Splenectomy			
Probability of complete response at 5–20 years	0.60 (0.60–0.885)	β-PERT (0.48 0.72)	Kojouri et al.^10^ Akwari et al.^11^ Kumar et al.^12^ Vianelli et al.^13^ Palandri et al.^14^ Mikhael et al.^15^
Probability of perioperative laparoscopic splenectomy mortality	0.002	β-PERT (0.002, 0.01)	Luu et al.^16^
Probability of perioperative open splenectomy mortality	0.01		Kojouri et al.^10^
Probability of infection requiring hospitalization s/p splenectomy, yearly	0.001385 (0.000746–0.001385)	β-PERT (0.000746, 0.001662)	Kyaw et al.^17^
Probability of overwhelming postsplenectomy death	0.70 (0.50–0.70)	β-PERT (0.50, 0.70)	Luu et al.^16^
Probability of thrombosis (venous and arterial), yearly	0.017	β-PERT (0.014, 0.020)	Ruggeri et al.^18^
Probability of accessory splenectomy	0.09	β-PERT (0.072, 0.108)	Akwari et al.^11^
Assumed probability of complete response s/p accessory splenectomy	0.00	-	Assumed
Annual cost of most expensive direct oral anticoagulant (indefinite use for postsplenectomy thrombosis)	$6000	γ (25, 240)	GoodRx^19^
Cost of laparoscopic splenectomy, vaccination, and accessory spleen imaging	$22 442	γ (25, 898)	Healthcare Bluebook^20^ GoodRx^19^ MDSave^21^ Chaturvedi et al.^22^
Mean cost of septic shock hospitalization	$42 285	γ (0.48, 87 376)	Paoli et al.^23^
Rituximab (1 cycle)			
Probability of overall response at 1 year	0.502 (0.38–0.625)	β-PERT (0.38, 0.625)	Godeau et al.^24^ Patel et al^25^ Ghanima et al.^26^ Arnold et al.^27^
Probability of overall response at 2 years	0.322 (0.310–0.333)	β-PERT (0.258, 0.386)	Godeau et al.^24^ Patel et al.^25^
Probability of overall response at 5 years	0.21	β-PERT (0.168, 0.252)	Patel et al^25^
Assumed probability of adverse events from rituximab	0.00	-	Assumed
Cost of rituximab (1 cycle)	$32 604	γ (25, 1304)	Goshua et al.^28^
TRA therapy			
Probability of initial overall response, assumed to be sustained as long as patient on therapy	0.80	β-PERT (0.64, 0.96)	Cuker and Neunert^29^
Probability of successful discontinuation after 2 years, assumed for rest of lifetime	0.28 (0.224–0.336)	β-PERT (0.224, 0.336)	Marshall et al.^30^
Assumed probability of adverse events from TRA	0.00	-	Assumed
Cost of TRA (yearly)	$113 976	γ (180, 634)	Chaturvedi et al.^22^ GoodRx^19^

Abbreviations: ITP, immune thrombocytopenia; TRA, thrombopoietin receptor agonist.

**TABLE 2:** Baseline cost-effectiveness analysis and probabilistic sensitivity analysis

#	Treatment strategy	Aligns with guidelines?	Cost ($ USD)	QALYs	ICER ($ USD per QALY)	Net monetary benefit ($ USD)	95% Credible interval (net monetary benefit)
5	Splenectomy → R → TRA	No	376 350	12.08	-	1 983 417	[1 831 036–2 061 149]
4	R → splenectomy → TRA	Yes	387 305	12.10	529 291	1 976 504	[1 829 135–2 053 408]
1	Splenectomy → TRA → R	Yes	472 118	12.13	3.0 million	1 897 261	[1 726 838–1 992 321]
3	R → TRA → splenectomy	Yes	794 637	12.14	Dominated	1 576 466	[1 388 454–1 696 599]
6	TRA → splenectomy → R	No	1 061 539	12.30	3.5 million	1 340 559	[1 111 919–1 487 970]
2	TRA → R → splenectomy	Yes	1 063 416	12.28	Dominated	1 334 003	[1 108 413–1 696 599]

Abbreviations: ICER, incremental cost-effectiveness ratio; QALY, quality-adjusted life-year; R, rituximab; TRA, thrombopoietin receptor agonist (romiplostim or eltrombopag); USD, United States Dollar.

## Data Availability

Data availability statement: The data that support the findings of this study are available on request from the corresponding author. The data are not publicly available due to privacy or ethical restrictions.
