# Supplementary material for: Cost‐effectiveness of second‐line therapies in adults with chronic immune thrombocytopenia
Source: Am J Hematol. 2022 Feb 24;98(1):122–30. doi: 10.1002/ajh.26497 (PMC9365880; doi:10.1002/ajh.26497)
Supplement: Supplementary file 1 — Figure S1 Markov schematic. Shown are the Markov schematics for the six treatment strategies in chronic ITP. Strategies #1–4 are in alignment with ASH guidelines. Strategies #5–6 are not in alignment with ASH guidelines. Red color bracket represents ongoing annual risk of postsplenectomy thrombosis and sepsis. TRA = thrombopoietin receptor agonist Table S1. One‐way deterministic sensitivity analyses. Red font represents parameter value corresponding to maximum ICER. All strategies were compared to the cost‐effective strategy #5. TRA = thrombopoetin receptor agonist, R = rituximab, ICER = incremental cost‐effectiveness ratio; USD = United States Dollar [file AJH-98-122-s001.pdf]

Supplementary Figure 1. Markov schematic. Shown are the Markov schematics for the six treatment strategies in chronic ITP. Strategies #1-4 are in alignment with ASH guidelines. Strategies #5-6 are not in alignment with ASH guidelines. Red color bracket represents ongoing annual risk of post-splenectomy thrombosis and sepsis. TRA = thrombopoietin receptor agonist

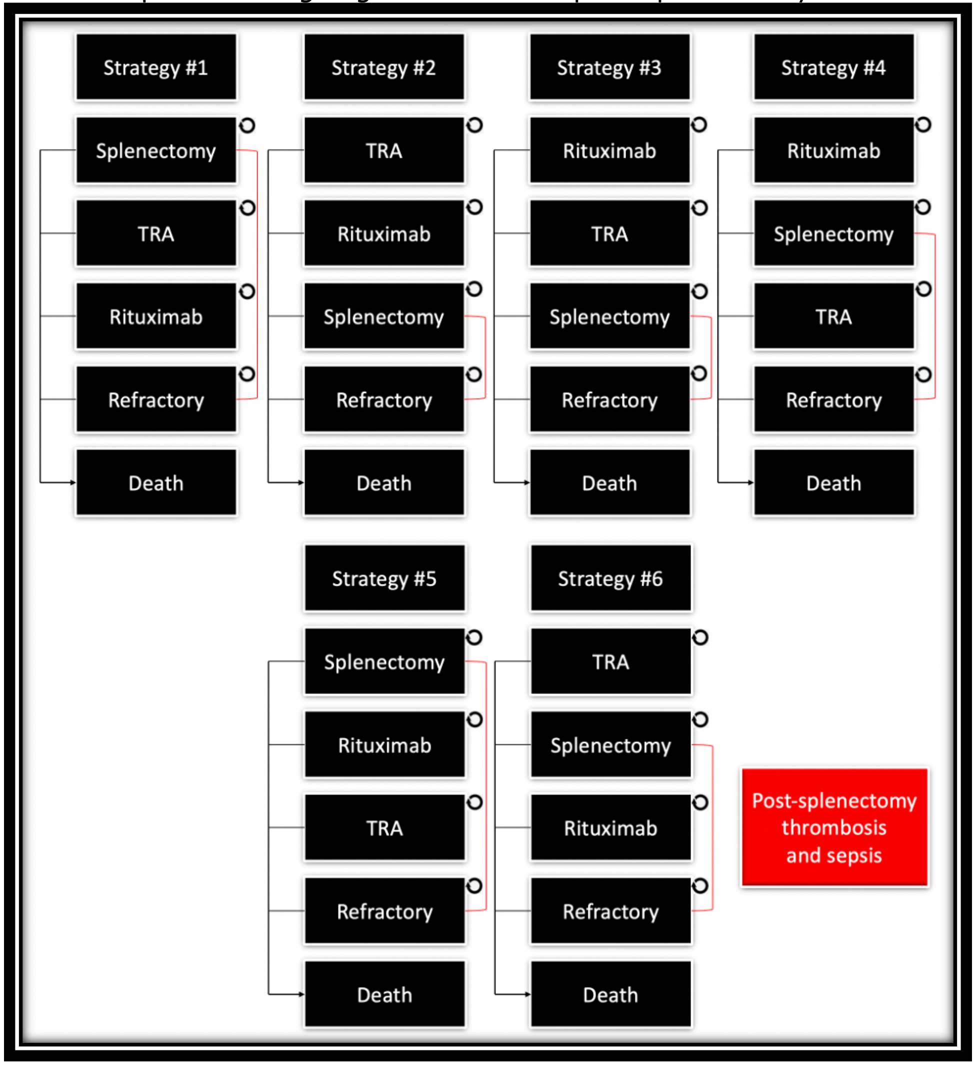

Supplementary Table 1. One-way deterministic sensitivity analyses. Red font represents parameter value corresponding to maximum ICER. All strategies were compared to the cost-effective strategy #5. TRA = thrombopoetin receptor agonist, R = rituximab, ICER = incremental cost-effectiveness ratio; USD = United States Dollar

**#1 (splenectomy -> TRA-> R) vs #5 (splenectomy -> R -> TRA) ICER $1.9 million**		
	Parameter Range	ICER Range ($ USD, millions)
Utility of disease state	0.592-0.888	1.2-4.8
Probability of overall response at 1 year (rituximab)	0.38-0.625	1.4-2.6
Utility of well state	0.900-0.987	1.9-3.0
Cost of TRA	$91,881-136,771	1.5-2.4
Probability of overall response at 2 years (rituximab)	0.266-0.400	1.6-2.4
Probability of overall response at 5 years (rituximab)	0.168-0.252	1.6-2.4
Probability of persistent overall response (TRA)	0.64-0.96	1.8-2.2
**#2 (TRA-> R -> splenectomy) vs #5 (splenectomy -> R -> TRA) ICER $3.6 million**		
Parameter	Parameter Range	ICER Range ($ USD, millions)
Probability of complete response at 5-20 years (splenectomy)	0.48-0.72	2.5-5.3
Probability of persistent overall response (TRA)	0.64-0.96	3.0-5.0
Utility of disease state	0.592-0.888	2.8-4.8
Cost of TRA	$91,881-136,771	2.8-4.3
Probability of perioperative mortality	0.002 (lap),<br>0.01 (open)	3.6,<br>2.5
Utility of well state	0.90-0.987	3.6-4.4
**#3 (R -> TRA -> splenectomy) vs #5 (splenectomy -> R -> TRA) ICER $7.2 million**		
Parameter	Parameter Range	ICER Range ($ USD, millions)
Probability of complete response at 5-20 years (splenectomy)	0.48-0.72	3.2-58.2
Probability of infection requiring hospitalization s/p splenectomy	7.5–16.6 per 100 person-years	2.2-5.6
Probability of persistent overall response (TRA)	0.64-0.96	5.0-18.9
Utility of disease state	0.592-0.888	4.6-16.2
Probability of overwhelming post-splenectomy death	0.50-0.70	7.2-12.4
Probability of overall response at 1 year (rituximab)	0.38-0.625	5.4-9.9
Probability of perioperative mortality	0.002 (lap),<br>0.01 (open)	7.2,<br>3.0
Probability of overall response at 2 years (rituximab)	0.266-0.400	5.8-9.0
Cost of TRA	$91,881-136,771	5.7-8.7
Probability of overall response at 5 years (rituximab)	0.168-0.252	6.0-8.8
Utility of well state	0.90-0.987	6.2-7.2

#4 (R -> splenectomy -> TRA) vs #5 (splenectomy -> R -> TRA) ICER $529,291		
Parameter	Parameter Range	ICER Range ($ USD, in hundreds of thousands)
Probability of overall response at 1 year (rituximab)	0.38-0.625	245-2764
Probability of infection requiring hospitalization s/p splenectomy	7.5–16.6 per 100 person-years	403-2096
Utility of disease state	0.592-0.888	320-1534
Probability of overall response at 2 years (rituximab)	0.266-0.400	300-1298
Probability of overall response at 5 years (rituximab)	0.168-0.252	316-1134
Cost of rituximab (1 cycle)	$26,083-39,125	330-729
Probability of overwhelming post-splenectomy death	0.50-0.70	529-973
Probability of perioperative mortality	0.002 (lap),<br>0.01 (open)	529,<br>238
Cost of laparoscopic splenectomy, vaccination, and accessory spleen imaging	$17,954-26,930	466-592
**#6 (TRA -> splenectomy -> R) vs #5 (splenectomy -> R -> TRA) ICER $3.2 million**		
Parameter	Parameter Range	ICER Range ($ USD, millions)
Probability of complete response at 5-20 years (splenectomy)	0.48-0.72	2.2-4.6
Utility of disease state	0.592-0.888	2.4-4.6
Cost of TRA	$91,881-136,771	2.5-3.8
Utility of well state	0.90-0.987	3.2-4.1
Probability of infection requiring hospitalization s/p splenectomy	7.5–16.6 per 100 person-years	2.9-3.8
Probability of perioperative mortality	0.002 (lap),<br>0.01 (open)	3.2,<br>2.3
Probability of persistent overall response (TRA)	0.64-0.96	2.9-3.5
